# The Challenges of Designing and Implementing Clinical Trials With Broccoli Sprouts… and Turning Evidence Into Public Health Action

**DOI:** 10.3389/fnut.2021.648788

**Published:** 2021-04-29

**Authors:** Jed W. Fahey, Thomas W. Kensler

**Affiliations:** ^1^Department of Medicine, Division of Clinical Pharmacology, Johns Hopkins School of Medicine, Baltimore, MD, United States; ^2^Department of Psychiatry and Behavioral Sciences, Johns Hopkins School of Medicine, Baltimore, MD, United States; ^3^Department of Pharmacology and Molecular Sciences, Johns Hopkins School of Medicine, Baltimore, MD, United States; ^4^Department of Nutrition and Food Studies, College of Health and Human Services, George Mason University, Fairfax, VA, United States; ^5^Translational Research Program, Fred Hutchinson Cancer Research Center, Seattle, WA, United States; ^6^Department of Environmental Health and Engineering, Johns Hopkins Bloomberg School of Public Health, Baltimore, MD, United States

**Keywords:** sulforaphane, glucoraphanin, myrosinase, pharmacokinetics, biomarkers, safety, efficacy, dietary supplement

## Abstract

Broccoli sprouts are a convenient and rich source of the glucosinolate glucoraphanin, which can generate the chemopreventive agent sulforaphane through the catalytic actions of plant myrosinase or β-thioglucosidases in the gut microflora. Sulforaphane, in turn, is an inducer of cytoprotective enzymes through activation of Nrf2 signaling, and a potent inhibitor of carcinogenesis in multiple murine models. Sulforaphane is also protective in models of diabetes, neurodegenerative disease, and other inflammatory processes, likely reflecting additional actions of Nrf2 and interactions with other signaling pathways. Translating this efficacy into the design and implementation of clinical chemoprevention trials, especially food-based trials, faces numerous challenges including the selection of the source, placebo, and dose as well as standardization of the formulation of the intervention material. Unlike in animals, purified sulforaphane has had very limited use in clinical studies. We have conducted a series of clinical studies and randomized clinical trials to evaluate the effects of composition (glucoraphanin-rich [± myrosinase] vs. sulforaphane-rich or mixture beverages), formulation (beverage vs. tablet) and dose, on the efficacy of these broccoli sprout-based preparations to evaluate safety, pharmacokinetics, pharmacodynamic action, and clinical benefit. While the challenges for the evaluation of broccoli sprouts in clinical trials are themselves formidable, further hurdles must be overcome to bring this science to public health action.

## Introduction

### Why Broccoli Sprouts?

Simply put, broccoli sprouts, and sprouts in general—less frequently called microgreens or even seedlings—are an excellent source of protein, vitamins, minerals, fiber, and especially phytochemicals. Phytochemicals are the compounds that are present in plants at very low levels compared to the proteins, carbohydrates, fats, and fiber that make up the bulk of most living

organisms. Phytochemicals are by-and-large made by the plants for their own protection or to give them an advantage in the environment in which they live. These 50,000 or so compounds have recently been called “the dark matter of nutrition” and are described as being “largely invisible to both epidemiological studies, as well as to the public at large” (1). They include colors (pigments), scents, and various compounds with antibiotic or other defensive activities. Thus, sprouts are in essence a microcosm of the larger plants which they will grow up to become, but they are fresh, extremely inexpensive and incredibly fast to grow (2). Anybody can grow them indoors, anywhere there's a square foot or so of free space, and commercially grown sprouts are widely available, though sprout sources, availability, or sprouting methods will not be part of this review.

### …But Really…

**Scheme 1:**
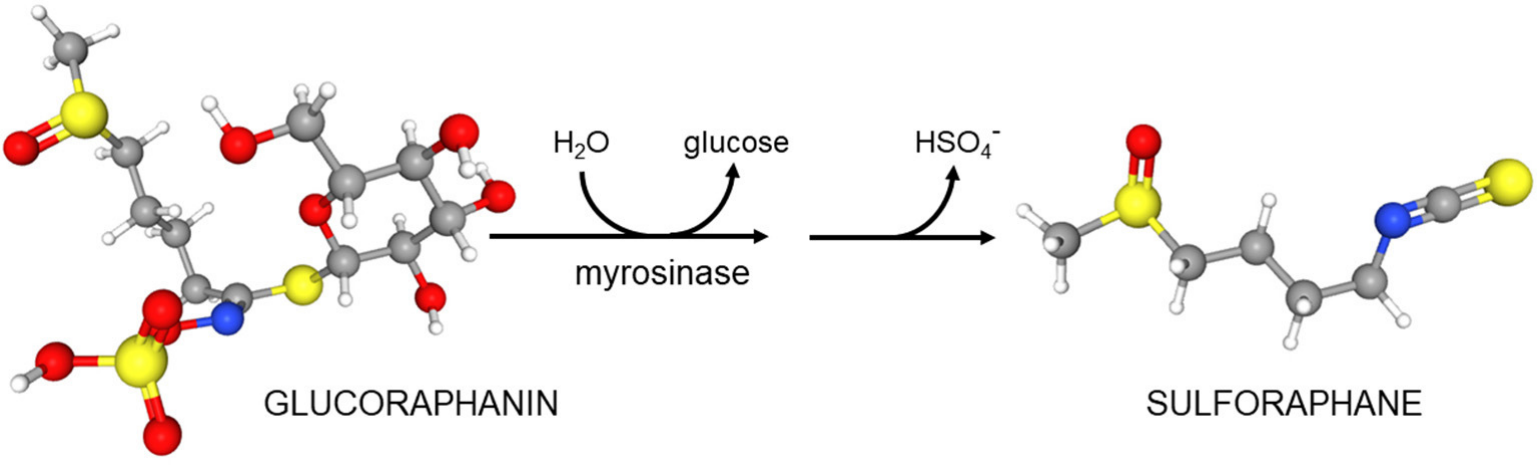


These considerations did not guide our development of broccoli sprouts as a novel protective and perhaps even therapeutic food. Rather, development was guided by epidemiology suggesting a protective effect of cruciferous vegetables including broccoli against a variety of cancers. In 1992, guided by bioassays, Paul Talalay and Yuesheng Zhang at The Johns Hopkins University School of Medicine identified sulforaphane in broccoli and determined its very potent cancer preventative action (3, 4). In 1993 one of us (JWF) joined Talalay's team to run a new lab called the Brassica Chemoprotection Laboratory. The initial mission of this lab was to identify “better broccoli”—a plant with more sulforaphane. We started on the eastern shore of Maryland growing broccoli in the field and bringing it back to the lab in Baltimore for analyses. Early discoveries were that there was a wide range of “potencies”—defined as the capacity of these extracts to induce carcinogen detoxication or “chemoprotective enzymes” (e.g., Nqo1) in a rapid, cell culture-based bioassay. This potency was almost entirely derived from the glucosinolates (predominantly glucoraphanin, the precursor of sulforaphane) in the plant tissue (5). Furthermore, we concluded that that there is actually almost no sulforaphane in healthy broccoli, but it is all present as its inert precursor glucoraphanin and (as was previously known) an enzyme called myrosinase that converted precursor to product upon wounding of the plant (e.g., chewing by predators that include people) (Scheme 1). Although other products can accompany the production of sulforaphane, it is generally sulforaphane, the isothiocyanate, which predominates [reviewed in (6)].

**Figure 1:**
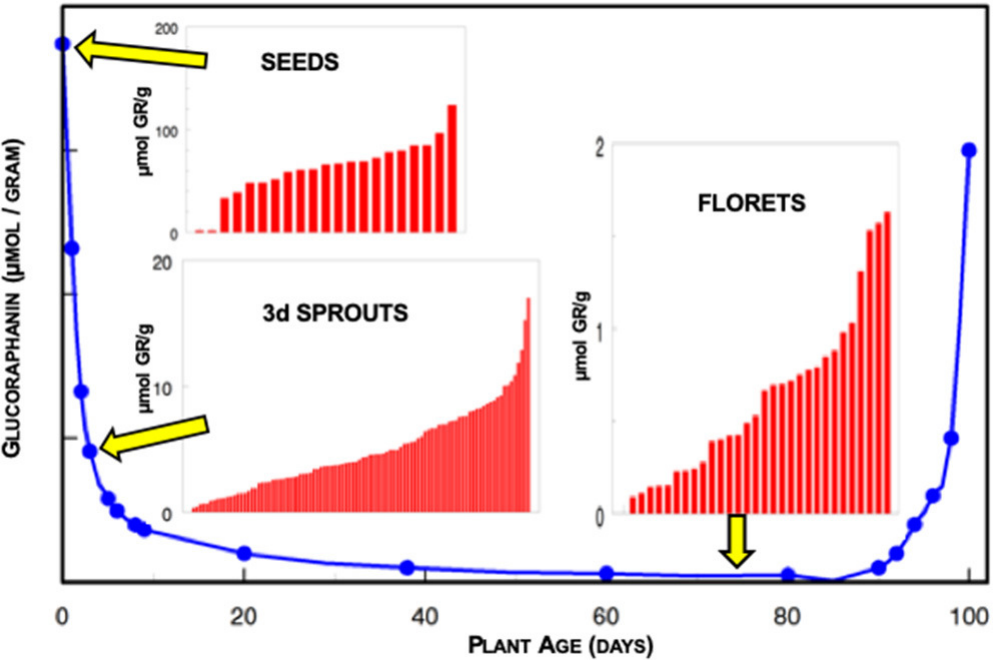
Glucoraphanin (GR) content of broccoli over the life course of the plant expressed on the basis of fresh weight. Inserts indicate the heterogeneity of glucoraphanin content in different cultivars of seeds (2–124 μmol GR/g), 3-day old sprouts (0.3–17 μmol GR/g), and market stage florets (<0.1–1.6 μmol GR/g) obtained commercially and measured in the laboratory of JWF. Note the differing scales for each of the insert histograms.

Genetics of the broccoli as well as the environment in which it was grown played major roles in determining the glucoraphanin levels of the plants (7–9). When the winter came we of course were unable to harvest broccoli from the farms, or even from the greenhouse at which we had been growing young plants so we started surface-sterilizing seeds, actually “disinfesting” them, and putting them on agar and growing small seedlings or sprouts in an incubator, on the 13th floor of Johns Hopkins Hospital's main building in Baltimore. We rapidly discovered that the seeds of broccoli had the highest concentrations of glucoraphanin (~100-fold higher than florets), and that one could reproducibly grow high-glucoraphanin sprouts from high-glucoraphanin seeds (Figure 1). Germination and the growth of the sprout merely diluted the glucoraphanin, up to a certain point somewhere in the range of 10–14 days old. We published our first paper on broccoli sprouts in 1997 (5). Subsequent to that, there was an explosion of work dealing with broccoli sprouts, glucoraphanin, myrosinase, sulforaphane, and animal and clinical studies utilizing this potent phytochemical system which had been aptly dubbed “the mustard oil bomb” (10), since this is a potent defensive system for the plant, against attack by pathogens such as fungi and insects. We humans have co-opted the plants' system for our defense.

### To the Clinic

With clinical pharmacologist Theresa Shapiro, we began investigating our ability to monitor broccoli (glucosinolate and sulforaphane) consumption and its metabolic fate in research subjects (11). We then performed our first interventions with broccoli sprouts, evaluating pharmacokinetics, acceptance, safety, and frankly, the reasonableness of using this new food source in clinical protocols (12, 13). These studies brought into sharp focus the issues that we would face developing robust “test articles” and acceptable placebos for such trials and this theme has persisted throughout all of our subsequent work.

While this plant-centric preventive research was going on at the JHU School of Medicine, one of us (TWK) was already evaluating clinical approaches to chemoprevention at the Bloomberg School of Public Health. Similar desires [to prevent cancer(s) and/or to intercept their further development once initiated and even before they become clinically evident] with a more pharmacologic approach, were also guided by epidemiology. The epidemiology in this case was that of contaminated foods and exposures (e.g., aflatoxins) that can lead to liver cancer, particularly in West Africa and in China. In 2001, following initial trials using an anti-schistosomal pharmaceutical in a particularly affected region of rural China, TWK decided to pivot to the newly emerging work with the phytochemical sulforaphane and attempt using broccoli sprouts as the intervention. We (TWK and JWF) together with a large team from Hopkins, the Qidong Liver Cancer Institute, and the Shanghai Cancer Institute, began a series of clinical studies to evaluate whether broccoli sprouts, or beverages or powders derived from them and rich in glucoraphanin, sulforaphane, or both, had demonstrable efficacy against biomarkers of unavoidable aflatoxin exposure (14). Over two decades, as liver cancer rates in our study region began to decline and air pollution exposures became a more prominent and widespread hazard throughout China (15, 16), we added biomarkers of lung toxicity and carcinogenesis to our outcomes of interest. In addition to evaluating safety, acceptability, bioavailability, and other pharmacokinetic parameters, we were able to successfully show favorable changes in biomarkers of both internal exposures to aflatoxin and air pollutants over multiple clinical trials, with multiple dose modalities, over many years, and most recently to demonstrate dose dependence (14, 17–20). Most of these trials have been reviewed elsewhere (21–23) and we will not dwell upon their outcomes herein.

**Figure 2:**
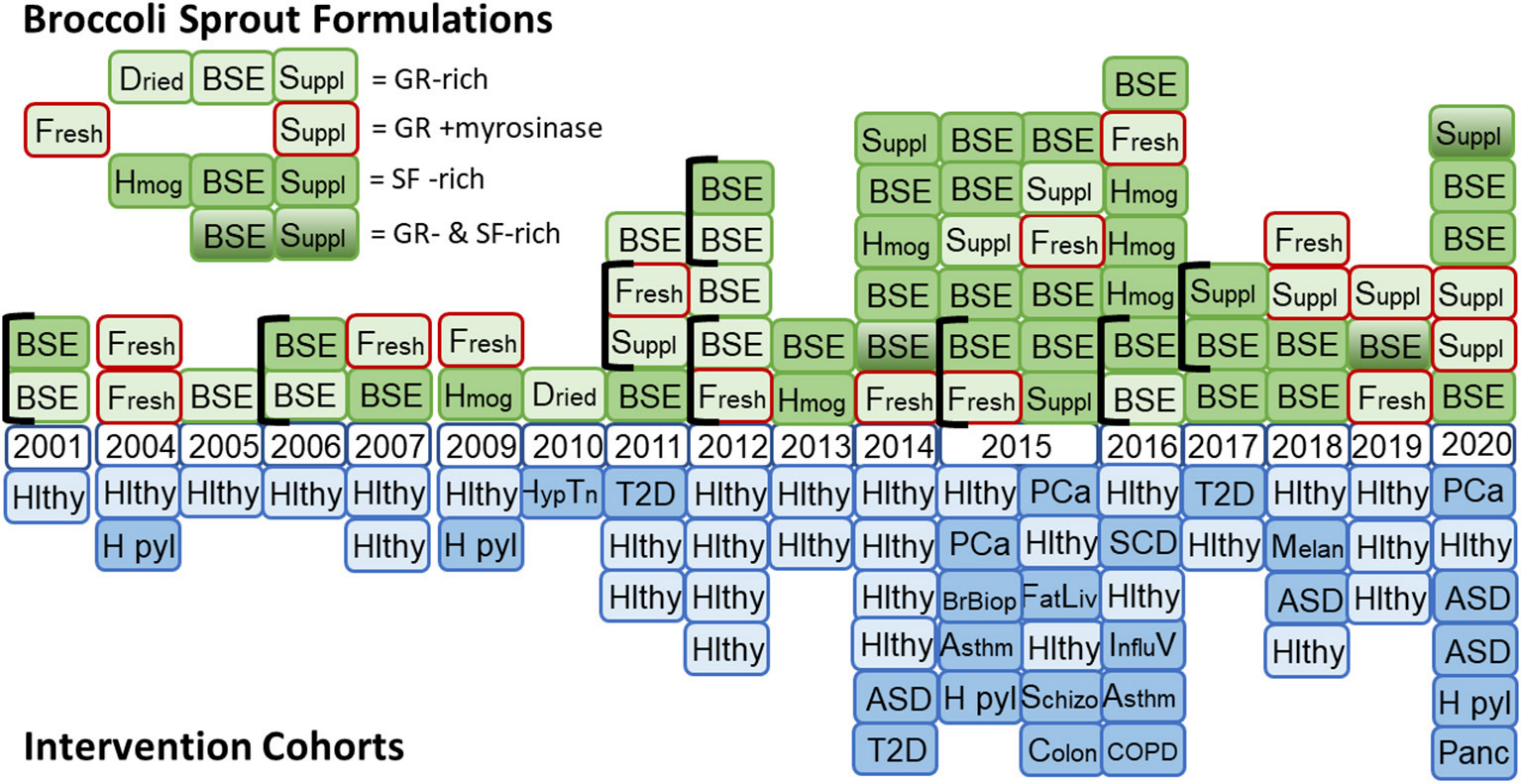
Timeline for the use of formulations of broccoli sprouts in clinical trials. (Top) Glucoraphanin (GR)-rich preparations included dried broccoli sprouts, hot water broccoli sprout extracts (BSE), and supplements formulated with freeze-dried broccoli sprouts or finely milled broccoli seeds to provide GR. Formulations enclosed with a red border included myrosinase (from either broccoli sprouts or daikon sprouts). Sulforaphane (SF)-rich formulations included fresh homogenates of broccoli sprouts, BSE treated with myrosinase or supplements including freeze-dried broccoli sprouts and finely milled broccoli seeds to provide myrosinase and GR. A few studies included blended formulations that included both GR-rich and SF-rich preparations. (Bottom) Mirrored against the study formulations are the study cohorts used with each formulation. Hlthy, healthy volunteers; H pyl, *Helicobacter pylori* infected participants; Hyp Tn, hypertensive participants; T2D, type 2 diabetes patients; ASD, autism spectrum disorder patients; PCa, prostate cancer patients; BrBiop, patients undergoing breast biospsy; Asthm, patients with asthma; FatLiv, patients with fatty liver; Schizo, schizophrenia patients; Colon, colon cancer patients; SCD, sickle cell disease patients; Influ V, influenza virus infected participants; COPD, chronic obstructive pulmonary disease; Melan, melanoma patients with multiple atypical nevi; Panc, patients with pancreatic cancer.

We and many others around the world, have developed an enormous body of laboratory and pre-clinical data, and a burgeoning body of clinical evidence addressing the potential that sulforaphane has, not only in the prevention of environmental carcinogenesis, but in the prevention or amelioration of a very large, diverse, and seemingly unrelated series of conditions. These conditions include autism spectrum disorder (ASD), schizophrenia, bacterial and viral infections, prostate, lung, breast, skin, and head and neck cancers, osteoarthritis, type 2 diabetes, sickle cell disease, fatty liver, and asthma. Presented in Figure 2 is the timeline of the conduct (publication) of clinical trials using broccoli sprout preparations (sulforaphane generating and/or glucoraphanin containing), which began in the late 1990s. This graph reflects the changing formulations used across time as well as a switch from predominantly healthy volunteer studies to those engaging with “at-risk” cohorts for multiple indications. Details of the clinical studies presented in this timeline are found in the following publications (11–15, 17–20, 24–69).

## Is it Just Sulforaphane?

Although almost all clinical studies have relied on broccoli sprout preparations or market stage broccoli, most of the data regarding possible mechanisms of action and efficacy were derived from cell culture and animal studies performed with pure sulforaphane. At the turn of the millennium, “pure sulforaphane” was clearly recognized as a drug by IRBs and the FDA, which precluded its entry into clinical trials in the absence of considerable (and expensive) safety data together with limited availability and difficulties in formulation. Food-based interventions were reviewed with much greater laxity in the “early days,” reliant upon notions of the intrinsic “safety” of commonly consumed foodstuffs. This perspective has now changed and all trials involving broccoli sprouts that seek endpoints reflecting efficacy rather than just pharmacokinetics, will likely require IND determinations from the FDA prior to conduct. A few sulforaphane-based pharmaceuticals are now under study (70).

It is pertinent to ask nonetheless whether there is bioequivalence in clinical experiments using broccoli sprouts, extracts of broccoli sprouts or seeds, or commercial dietary supplements containing same and neat sulforaphane itself. Thus, the contributions of any of a very large range of additional phytochemicals (e.g., flavonoids, anthocyanins, carotenoids) found in sprouts cannot be ruled out completely, and could prove beneficial. These potential phytochemical interactions have not been studied in a comprehensive fashion, although: (a) some studies of synergies and additive effects of other phytochemicals with sulforaphane have been undertaken (71); (b) drug-sulforaphane interactions have been studied (50, 72); and (c) a pilot study has been undertaken to assess the feasibility of a fully-powered study to examine the effects of a phytotherapeutic intervention (containing turmeric, resveratrol, green tea and broccoli sprouts) on PSA doubling time in men with biochemical recurrence with a moderate PSA rise rate (73). Much more needs to be learned regarding the extent to which phytochemical reductionism mimics or impedes clinical bioequivalence.

## The Need for Standardization and Validation of Broccoli Sprouts

This was a problem that we first highlighted a decade ago, and its need for resolution has changed very little since then (74). Investigators must provide chemical analyses of at the very least, the glucoraphanin, sulforaphane, and myrosinase content of the materials that they employ in clinical trials. Those studies that do not, may be basing any results which they report, on a faulty premise related to “dose” given. When using fresh broccoli sprouts, it is an absolute requirement that these values be measured and reported. The plants sourced and used for these trials will vary by pedigree (genotype), growth environment, and production characteristics (e.g., time-to-harvest, temperature, lighting) and this in turn has an effect on phytochemical content. The potential presence of food-borne pathogens, the sprout-associated microflora if one is administering fresh sprouts or a non-cooked preparation, is a huge concern. And, of course, screening for the presence of pesticides, heavy metals, and other unintended contaminants is associated with good manufacturing practices in both the pharmaceutical, food, and supplement industries, but this can easily escape the attention of investigators eager to use foods as a clinical intervention. Many clinical investigations that use dietary supplements take what is perceived to be an easy way out and merely report the contents as given by the manufacturer. This is fraught with problems in part because there are few commercial labs that can properly perform the analyses for glucoraphanin, myrosinase, and/or sulforaphane, nor do many of the manufacturers or “assemblers of supplement components” (i.e., vendors, encapsulators, and purveyors) understand the subtleties of stability and storage issues unique to these phytochemicals. Thus, clinical studies done with those non-analyzed materials have the potential to waste enormous amounts of money by invalidating or lessening the scientific value of studies using well-characterized broccoli sprout preparations.

## Transitions in Formulations—a Natural Evolution

For reasons, some of which we have itemized herein, there has been a transition from fresh broccoli sprouts to broccoli sprout extracts, to powders, capsules, and dietary supplements made from them. This is natural and logical and it stems from a variety of causes. First, it represents the evolution from a discovery of potent activity in a plant, to identification of the best part and stage of the plant in which to find that activity, to frustration at the difficulty of sourcing proper seeds of that plant, and reproducibly growing the proper sprouts at large scale in order to ensure delivery of a reproducible dose of the desired phytochemical.

Sulforaphane (the principal broccoli sprout phytochemical that is biologically active in human beings) is very unstable in combination with other organic materials, though relatively stable in pure (neat) form when refrigerated or frozen. Thus, initial “dosing” attempts utilized glucoraphanin, the biogenic precursor of sulforaphane, and the phytochemical which the plant too, maintains in storage vacuoles, at the ready to be activated by reaction with myrosinase. Glucoraphanin is easily extracted into aqueous solutions, and dried. Subsequent attempts to deliver an extract rich in sulforaphane utilized the myrosinase reaction to convert glucoraphanin-rich extracts to sulforaphane-rich extracts. For delivery of those products in the clinic it was necessary to maintain the lyophyilized extracts at −20°C for short periods of time, and even more ideal, at −80°C, a temperature at which they can be kept for many years. Alternatively, glucoraphanin was delivered along with active myrosinase, thus eliminating some of the issues of storage temperature as long as the product to be delivered to people was kept dry prior to use (ingestion).

Supplement manufacturers quickly adopted both glucoraphanin and glucoraphanin plus active myrosinase as delivery modalities, and as has already been apparent to clinical trialists, manufacturers have had far less success delivering stable sulforaphane in dietary supplements. Many such supplements that advertise to unwitting consumers their ability to do this, are making fraudulent claims because their products have very little or no sulforaphane left in them by the time they reach customers. Some clinical trialists assume that if using dietary supplements as their source of glucoraphanin or sulforaphane, manufacturer representations as to titer result in a certified product that requires no further testing or validation. These improperly tested products clearly don't deliver value to well-intentioned consumers, but lasting damage can be done to our understanding of the benefits of these compounds if clinical trials are written off as “negative,” based on faulty assumptions about dose. Providing sulforaphane or other phytochemicals to be tested in the form of a dietary supplement is probably an ideal solution for clinical trialists studying the actions of a specific phytochemical, but there is a very strong argument that it is too reductionist an approach to maintaining the human body in optimum healthspan. It ignores food matrix effects, cultural, sensory, satietal, social, and indeed optimum nutritional benefits of eating whole or even lightly processed foods.

## Selecting a Placebo

Development of an appropriate placebo for the unfolding series of broccoli (“sulforaphane”) trials that we and others have conducted has been a challenge. We opined on this challenge in the context of chemoprotection trials in some detail, a decade ago (74). Only limited progress has been made since then. To wit:

Initial trials utilized either fresh broccoli sprouts (33, 36), boiling water extracts of fresh broccoli sprouts (14), or freeze-dried extracts of broccoli sprouts that were either glucoraphanin-rich or sulforaphane-rich. These powders were placed in gel caps or given in minimal volumes of water, directly to subjects. Identifying placebos for fresh sprouts is exceedingly difficult and nobody has developed appropriate placebos, though alfalfa sprouts have been used in the clinic (36) in an attempt to provide a non-sulforaphane-containing vegetable placebo with similar organoleptic properties. Boiling water extracts of fresh broccoli sprouts presented a problem of balancing taste/flavor with the need to remove all traces of glucoraphanin. Fortunately, glucoraphanin is highly soluble and easily diluted away from the sprout mass on the first extraction with boiling water. By performing multiple boiling water extractions were we able to obtain a mildly colored, flavored, yet “vegetable” smelling extract that was essentially free of glucoraphanin (or sulforaphane) (14).

Powdered lyophilized extracts of sprouts were initially used in non-placebo controlled, small studies after re-dissolving in minimal volumes of water [e.g., (12, 26, 28, 47, 75)]. In other trials, the lyophilized powders were placed in gel caps, and controls were prepared using only microcrystalline cellulose, also placed in gel caps, and in some cases colored gel caps (e.g., blue, purple, green) were utilized to mask color differences (28, 54, 55, 60, 64). Masking was adequate, however, the odor of broccoli extracts that emanated from gel caps made discrimination of dose from placebo clear to anyone who was able to compare capsules.

Juice-based delivery approaches have enabled the development of better flavor misattribution strategies for broccoli sprout and seed extracts. They have enabled vehicle-alone placebos to be used more effectively (76). We have been able to move from a simple taste *masking* strategy for which we and others used dilute mango juice (17, 18, 52, 56), to one in which we used dilute pineapple and lime juice (19, 20) in an applied sensory evaluation technique that was scientifically developed to align subject expectations with sensory properties. Thus, the identification of pineapple and lime juice was based on the development of a complementary flavor profile, following extensive descriptive analysis of the taste profile of broccoli sprout extracts and a search for more “favorable” flavor notes that would lead to *misattribution* (rather than masking) of the broccoli flavor to other beverage components (76, 77). With this strategy there were no concerns on the part of the investigators that either there would be large-scale rejection of the doses and placebos, or that there would be discovery by the participants of which were dose and which were placebo beverages.

In the last decade, the development of reliable and well-standardized, tested, and vetted dietary supplements by private industry, has advanced our ability to effectively utilize these gel caps and tablets in clinical trials. Initial efforts to create placebos, however, have still been met with frustration.

The earliest supplement-based, placebo-controlled trials now published utilized a commercial product, a tablet containing both glucoraphanin and myrosinase along with maltodextrin and other typical tablet excipients. Used in a placebo-controlled trial with children with ASD who typically are extremely fussy and particular eaters, and who were projected not to be able to swallow these tablets, we anticipated that whatever placebos were prepared would need to be ground up using an inexpensive hand-held pill grinding device that we gave to all parents, and co-administered with the “food of choice” for that child. Preparation of a custom placebo was ultimately performed under contract by researchers at a school of pharmacy, and wound up being a research project in and of itself. Matching texture, formulation, visual appearance (speckled), and consistency was a costly and lengthy process even employing the assistance of an academic research pharmacy. However, masking for this long-term study was ultimately successful (67, 78). The supplement manufacturer eventually provided placebos for their tablets' use in other trials that have not yet been published (21).

Due to the odor of broccoli emanating from gel caps containing powdered extracts, masking remains an issue, and some supplement manufacturers are now exploring the use of odor-absorbing package inserts. Some investigators have used directly dried and milled broccoli sprouts. In these cases, development of a placebo has been confounded by issues of color. Thus, cornstarch colored with chlorophyll has been used for trials in which powder was the mode of dose delivery (79). The most imaginative solution to this problem was the use of chlorophyllin (a semi-synthetic chlorophyll derivative used widely for odor control in ostomy patients), which was spray-dried with maltodextrin to create a placebo (61). This too, however, presents certain problems and concerns that were not recognized and likely not problematic in the trial cited, but are potentially a problem in other disease prevention trials since chlorophyllin is an effective inducer of cytoprotective enzymes (80) and, in a randomized clinical trial in Qidong, China, modulated aflatoxin disposition (81).

## Study Cohorts: Pharmacokinetics, Biomarkers of Pharmacodynamic Action, and Other Outcomes in Clinical Trials

Nearly 70 clinical studies, ranging from small Phase 0 or 1 trials examining pharmacokinetics or early pharmacodynamic action to larger placebo-controlled Phase II trials examining endpoints reflecting clinical efficacy, have been conducted and published using broccoli sprout-derived preparations and formulations. While the majority of these studies have been conducted with healthy volunteers, a substantial number of trials have been conducted in at-risk participants with hypertension, type 2 diabetes, fatty liver, sickle cell disease, asthma, COPD, schizophrenia, ASD, influenza virus infections, *Helicobacter pylori* infections, as well as patients with precancerous lesions or cancers including melanoma, prostate, or pancreas (see Figure 2). The design and outcomes of most of these trials have been summarized by us in 2020 and earlier reviews (21–23). Clinical signatures of efficacy have been reported in settings of treatment of *H. pylori* infections (33, 36, 51), improved clinical scales of ASD (43, 54) or schizophrenia (30), serum glucose and insulin resistance measures in type 2 diabetic patients (48, 61, 79), reduced airway resistance in asthmatics (56), and declining PSA trajectories in prostate cancer patients (58). Of course, not all broccoli sprout-based trials in these and the other settings have reported positive clinical outcomes. While none of these studies provide a clear guidepost for unrestrained enthusiasm, continuing refinement of the science for optimizing admixture of proper formulations, doses, study cohorts, and endpoints are likely to define clear directions for the use of broccoli sprouts in health maintenance and disease prevention. Stronger clinical signatures of efficacy in carefully designed, conducted, and analyzed clinical trials, should they emerge, are likely to provide the scientific backbone necessary for enhanced consumer acceptance. There is an opportunity here for science rather than hype to add value to the conversation.

### Pharmacokinetics

Studies on the pharmacokinetics of sulforaphane in healthy volunteers were fostered by the development of rigorous analytical methods to measure the levels of sulforaphane and its metabolites in blood, plasma, urine, and tissues following administration of broccoli and broccoli sprout based preparations. The initial studies by Shapiro, Talalay and colleagues quantified these analytes (dithiocarbamates) collectively by cyclocondensation with 1,2-benzenedithiol, with sensitivity in the picomolar range (82). This highly sensitive, simple, and convenient method continues to be used to assess the bioavailability of sulforaphane across a spectrum of formulations. In addition, methods have been developed to analyze the individual metabolites following their separation by liquid chromatography coupled with tandem mass spectrometry (66, 83–85). Furthermore, the use of mass spectrometry coupled with stable isotope-labeled internal standards of sulforaphane [1-isothiocyanato-4-methyl-sulfinyl(1,1,2,2,3,3,4,4-2H8)butane] and its corresponding mercapturic acid pathway conjugates allows for quantitative, precise, sensitive, and specific analysis of sulforaphane and its metabolites in biospecimens (85).

With these analytical tools in hand, a number of pharmacokinetic studies have been conducted in humans (and rodents). Following oral administration of 200 μmol broccoli sprout isothiocyanates to four healthy human volunteers, the peak plasma dithiocarbamate concentration (C_max_) was 1.91 ± 0.24 μM 1 h after dosing, with half-life of 1.77 ± 0.13 h, and clearance of 369 ± 53 ml/min (82). A study in 20 participants administered 200 μmol sulforaphane as sulforaphane-rich powder in capsules reported a C_max_ of 0.7 ± 0.2 μM at 3 h, with a half-life of 1.9 ± 0.4 h for elimination of sulforaphane equivalents measured by mass spectrometry (27). Another study reported plasma dithiocarbamate levels of 0.92 ± 0.72 μM and mean epithelial-/stromal-enriched breast tissue dithiocarbamate concentration of 1.45 ± 1.12 and 2.00 ± 1.95 pmol/mg tissue for the right and the left breast, respectively, in eight healthy women undergoing reduction mammoplasty who had received a single dose of a broccoli sprout preparation delivering 200 μmol sulforaphane 1 h prior to surgery (47). In a double-blind randomized placebo-controlled trial in men presenting for prostate biopsy, plasma levels of 0.12 μM of sulforaphane, and its metabolites were detected after an intervention period of 4–8 weeks with two daily doses of 100 μmol sulforaphane administered 12 h apart (65). Collectively, these studies rather consistently indicate that sulforaphane reaches only low micromolar peak plasma concentrations and exhibits a short biological half-life in clinical trial settings. These pharmacokinetic parameters should invoke a modicum of caution for those interpreting cell culture-based studies of the myriad of potential mechanisms of action of sulforaphane, wherein log-higher concentrations are often used.

Administration of the precursor glucoraphanin vs. the bioactive sulforaphane in broccoli sprout preparations profoundly affects bioavailability as determined by measures of the urinary excretion of sulforaphane and its metabolites. Regardless of starting material, little sulforaphane *per se* is excreted in urine, rather glutathione conjugate derived metabolites (predominantly sulforaphane *N*-acetyl cysteine) dominate (85). A study in healthy subjects who received single oral doses of broccoli sprout extracts containing the equivalent of 111 μmol of glucosinolates or isothiocyanates showed cumulative urinary dithiocarbamate excretion of 88.9 ± 5.5 and 13.1 ± 1.9 μmol for the isothiocyanate and the glucosinolate preparation, respectively (12). This study further revealed that for the isothiocyanate preparation, excretion was consistent, and linear over a 25–200 μmol dose range, whereas for the glucosinolate preparation, excretion was highly variable among individuals. These observations are in close agreement with results from a randomized, placebo-controlled, double-blind Phase I clinical trial, in which isothiocyanate (25 μmol)- or glucosinolate (25 μmol or 100 μmol)-rich preparations were orally administered to three cohorts of three healthy human subjects at 8-h intervals for 7 days (13).

The finding that compared to isothiocyanates, oral administration of glucosinolates results in slower elimination, lower bioavailability, and greater inter-individual variation in excretion was further strengthened by a larger (50 participants) crossover clinical trial that involved 5-day baseline period followed by daily administration of broccoli sprout beverages delivering either glucoraphanin or sulforaphane for 7 days, followed by a 5-day washout period, and then a 7-day administration of the opposite intervention (18). With both formulations, essentially all ingested sulforaphane equivalents were excreted within 24 h of dosing. However, elimination was much more rapid with sulforaphane as the starting material than with glucoraphanin. In this study the whole-body half-lives of sulforaphane and glucoraphanin from beverages were 2.4 and 7.3 h, respectively. Bioavailability was judged to be about 70% with sulforaphane-rich beverage and only 5% with glucoraphanin-rich beverage (but ranging from 1 to 45% across individuals). Using fecal sample collections from five subjects with high 24-h urinary excretion profiles (“high converters”) and five subjects with low excretion profiles (“low converters”), it was found that *ex vivo*, the degradation of glucoraphanin was greater in cultures of fecal bacteria derived from the “high converters” in comparison to the “low converters” (86). These observations were consistent with earlier work showing that mechanical cleansing or antibiotic treatment greatly reduced the glucosinolate conversion in healthy human subjects (11) and indicated that the gastrointestinal microflora represents a critical factor in determining the extent of glucosinolate hydrolysis. In addition to the inter-individual variations, there are also diurnal variations in the conversion of glucosinolates to dithiocarbamates, whereby conversion is greater during the day (75). By contrast, the conversion of isothiocyanates to dithiocarbamates is higher during the night.

Formulation, which in turn reflects how broccoli sprout extracts are prepared (e.g., with or without exogenous myrosinase-catalyzed hydrolysis of glucoraphanin), strongly affects bioavailability, both in terms of inter- and intra-individual consistency with repeated doses. Using a dietary supplement formulation of glucoraphanin (from boiled water extracts of broccoli seeds) plus myrosinase (from freeze-dried sprouts) in tablet form, we observed a median 20% bioavailability with greatly dampened inter-individual variability (21). Fahey et al. (46) have observed approximately 35% bioavailability with this supplement in a different population. While consistency in bioavailability (and product stability) is improved with supplements relative to some other formulations, insufficient studies have been published to date to infer improved efficacy.

### Biomarkers of Pharmacodynamic Action

**Figure 3:**
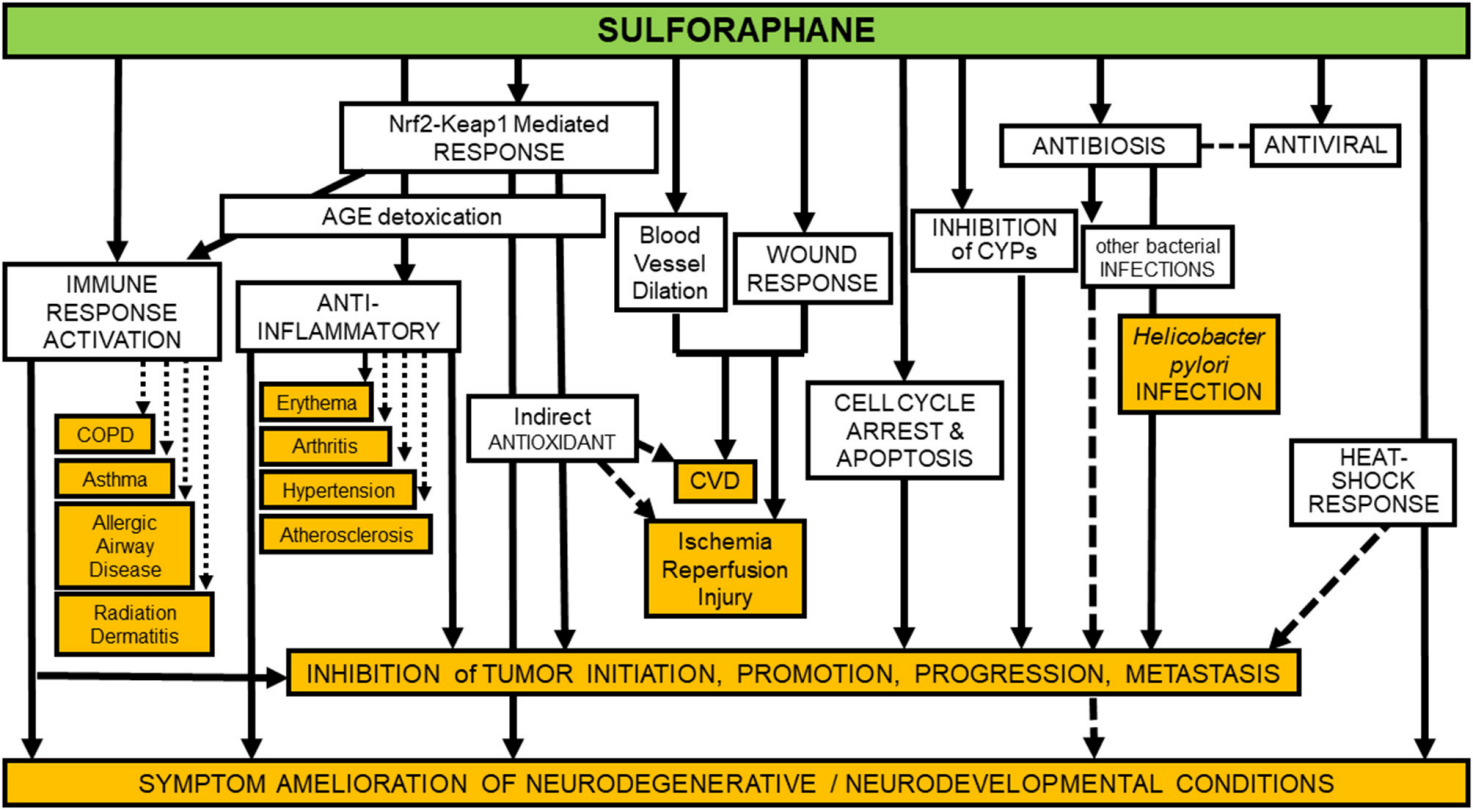
Identified preventive and protective molecular mechanisms of sulforaphane are manifold. Unfilled boxes show some of the more robustly documented mechanisms, whereas orange-filled boxes indicate some of the more well-documented diseases, syndromes, and conditions against which sulforaphane's efficacy has been assessed in clinical, animal, and *in-vitro* studies. Mechanisms involved in inhibition of tumor initiation, promotion, progression and metastasis (second box from bottom of figure) are too numerous to fully itemize on a simplistic diagram such as this, but in addition to those indicated by arrows directed toward the box, they include: *inhibition* of NFκB, HDACs, Pgp, MRP-1, BCRP, STAT3, MEKK1 activity, AP-1 DNA binding, and tubulin polymerization; *degradation* of α and β-tubulin; *down-regulation* of cyclin B1, cdk1, cdc25B, cdc25C, HIF, VEGF, VEGF receptor, MMP-2, and MMP-9; *modulation* of Bcl-2 family proteins; and *activation* of caspases (87).

Unlike many drugs discovered or initially developed in academic laboratories, the clinical development of broccoli-derived sulforaphane continues to be sustained by an increasing number of academic laboratories. Studies are funded principally through investigator-initiated grants from national funding agencies. As such, there is no concerted clinical development plan for sprout-based interventions, as would be seen with new chemical entities in the pharmaceutical industry. While this scientific approach has impeded the focused clinical evaluation of sulforaphane, it has conversely led to a rich variety of studies attempting to determine pharmacodynamic action. Inasmuch as sulforaphane was first isolated and characterized from broccoli through a bioassay directed screen of the induction of Nqo1 activity in murine Hepa 1c1c7 cells—an action mediated through Nrf2—this transcription factor has become a major focus of interest in the clinical setting (23). Induction of NQO1—transcripts or activity—have been measured in surrogate cells such as peripheral blood mononuclear cells and in target tissues following short-term broccoli sprout-based interventions. Indeed, NQO1 induction has been the most consistently observed response (22). Induction levels *in vivo* have been modest typically (~2-fold). Recent efforts have examined broader ranges of genes, using either targeted (e.g., other candidate genes) or untargeted (e.g., metabolomic) methods, to develop induction “signatures” (67). This approach, while nascent, looks promising. Additional biomarker candidates, probably reflecting engagement with NRF2 signaling, have also been explored. Oxidative stress biomarkers such as oxidative products of lipids, proteins, and DNA, as well as inflammatory mediators have been measured. We have relied on urinary biomarkers monitoring detoxication metabolites of environmental carcinogens in settings of unavoidable exposures to air pollutants (e.g., aldehydes, polycyclic aromatic hydrocarbons, and benzene) and dietary contaminants (e.g., aflatoxins) in our China trials. These biomarkers are consistently elevated with broccoli sprout-based interventions, although extrapolation of biomarker change to extent of cancer risk reduction has not been realized. Lastly, it is well-recognized that sulforaphane may interact with multiple cell signaling pathways and other targets independent of NRF2 (87–89). Multiple studies have focused on pathways affecting cancer development and progression. Additional promising outcomes in trials with sulforaphane-rich preparations have centered on modulation of epigenetic regulators such as histone deacetylase and histone acetyltransferase activities (90). Figure 3 presents an overview of possible targets and modes of action for sulforaphane, largely observed in preclinical studies, but recapitulated with some degree of consistency in clinical trials.

### Dose-Limiting Toxicities

Few clinical trials have provided a clear accounting of adverse events (AEs), likely signifying a general absence. The range of AEs we have observed in our trials included issues with taste (bitterness), gastrointestinal irritation, gas and flatulence, diarrhea, and vomiting. These outcomes were strongly influenced by formulation, with sulforaphane-containing sprout beverage preparations less well-tolerated than ones with glucoraphanin. Masking or “misattribution” of the taste as well as administering sprout powders within capsules or tablets, as done in more recent trials, has served to ameliorate the reporting of AEs. Dose, of course, was also an important determinant. However, in our experiences only a few grade I toxicities have been observed: 2 out of 50 participants receiving 150 μmol sulforaphane-rich beverage daily for 14 days and 6 out of 142 participants receiving a blended beverage containing 40 μmol of sulforaphane and 600 μmol of glucoraphanin for 84 days (14, 18–20). Participants receiving up to 800 μmol glucoraphanin reported no AEs (14, 18). Collectively, in just these four reported trials, close to 15,000 doses of broccoli sprout beverages were consumed by study participants. There were no alterations in standard clinical chemistry tests (i.e., liver and kidney function) amongst pre- and post-intervention measures in any of these studies. Based upon other reports in the literature, we estimate that 450 μmol of sulforaphane as a beverage or soup exceeds a tolerable level for healthy individuals and that a maximum tolerated dose may be 200 μmol or lower (21, 50).

An early study by Shapiro et al. (13) examined 32 types of hematology and chemistry tests, including thyroid function (TSH, T3, and T4) tests. Altered thyroid function is a potential concern that is occasionally raised in association with crucifer-based interventions, given the presence of low levels of goitrogens. There were no significant or consistent thyroidal toxicities when broccoli sprout derived isothiocyanate (25 μmol) or glucosinolate-rich (25 or 100 μmol) preparations were administered orally at 8-h intervals for 7 days in a small study. We followed up on this potential concern by analyzing biochemical measures of thyroid function and thyroid autoimmunity in 45 female participants in a randomized, placebo controlled clinical trial at baseline and after 84 days of beverage administration (40 μmol sulforaphane and 600 μmol of glucoraphanin) (91). Serum levels of thyroid-stimulating hormone, free thyroxine, and thyroglobulin were not affected by the treatment, nor was the thyroid autoimmunity status of the participants.

### Selecting a Dose: Importance of Internal Dose

**Figure 4:**
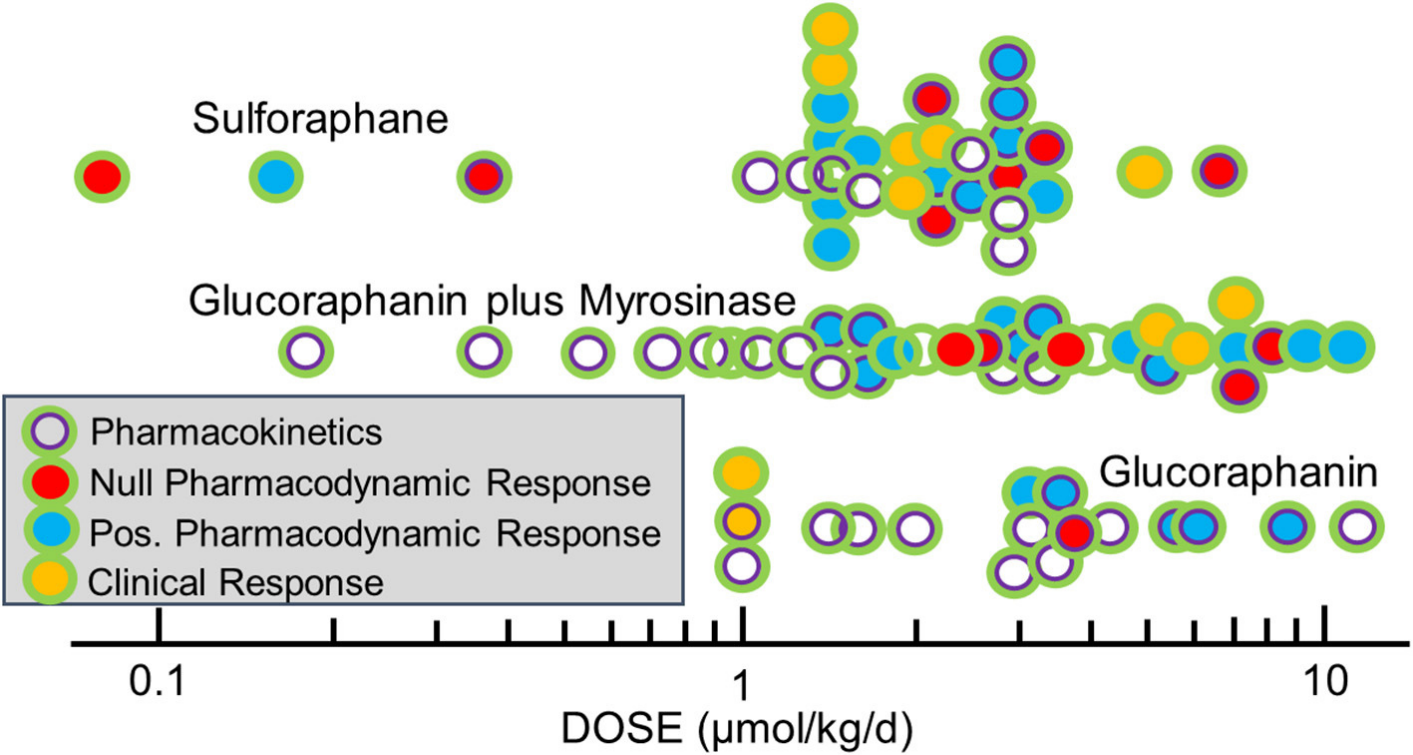
Doses of glucoraphanin (GR)-rich, GR+myrosinase, or sulforaphane (SF)-rich broccoli sprout preparations used in clinical trials. Pharmacodynamic biomarkers included measures of NRF2 target genes, gene expression/function, inflammation, oxidative stress, carcinogenesis, and/or metabolomics. Doses were calculated as listed in Yagishita et al. (21) and outcome measures from classifications in Yagishita et al. (22).

The selection of dose is complicated by the very different bioavailability of sulforaphane when administered in the precursor form of glucoraphanin and when given as sulforaphane itself, as discussed earlier. Thus, simple reporting of administered dose of glucoraphanin and/or sulforaphane can be a poor measure of the bioavailable/bioactive dose of sulforaphane achieved internally. As a consequence, we propose that the excreted amount of sulforaphane metabolites (sulforaphane + sulforaphane cysteine-glycine + sulforaphane cysteine + sulforaphane N-acetylcysteine) in urine over 24 h (2–3 half-lives), which is a measure of “internal dose,” provides a more revealing and likely consistent view of the delivery of sulforaphane to study participants. In turn, use of “internal dose” metrics will facilitate optimization of the linkage between formulation, dose, and schedule with determinants of efficacy and, importantly, allow more facile comparisons of results between different clinical trials. As an example, we find the 24-h recovery of sulforaphane metabolites in urine following dosing to be highest (~110 μmol) following administration of 150 μmol of sulforaphane-rich beverage; ~60 μmol following administration of a sulforaphane-glucoraphanin blend (40 and 600 μmol, respectively); ~25 μmol following administration of 800 μmol of glucoraphanin-rich beverage and ~25 μmol following administration of a dietary supplement tablet containing 150 μmol glucoraphanin plus myrosinase (21). In this series of trials, formulations that provided a 24-h internal dose of >~25 μmol sulforaphane metabolites evoked significant enhancements of carcinogen detoxication in study participants; lower internal doses did not. Figure 4 details the administered doses, normalized to μmol/kg/day used in published clinical trials of sulforaphane-rich, glucoraphanin-rich and glucoraphanin plus myrosinase preparations. Author-reported significant modulations of pharmacodynamic biomarkers or clinical endpoints were observed for all formulations, for the most part at external doses >1 μmol/kg/day or about 70 μmol per person. From this baseline, efficacy clusters largely between 1 and 3 μmol/kg/day with sulforaphane. Highlighting the poorer and more variable bioavailability seen with glucoraphanin, effective doses range from 1 to 10 μmol/kg/day for glucoraphanin plus myrosinase and neat glucoraphanin. Not all clinical trials have independently confirmed the external doses administered nor have they provided measures of internal dose. Adopting protocols to more rigorously assess internal dose across trials (together with accurate assessments of administered doses) will facilitate comparisons of test articles as well as study outcomes.

## Making the Science Practical: Can We Expand From Broccoli Sprout Clinical Trials to Population-Based Strategies?

Moving beyond the here and now is always a challenge. With novel foods about which an exciting marketing story can be spun, stakeholder companies stand to profit after investing in the development of packaging, advertising, logistics, sourcing, and perhaps even [plant or animal] growing. Sometimes mass-market food-, science-, or health reporting helps by calling a new food a “superfood” or giving it some other attractive and timely cachet (92). With new drugs, the potential to cure, mitigate, or prevent a disease, syndrome, or condition, in many cases requires tremendous investment to develop the safety and efficacy profiles of that drug and then the exploiting of an existing and well-established marketing stratagem to get it into use.

**Figure 5:**
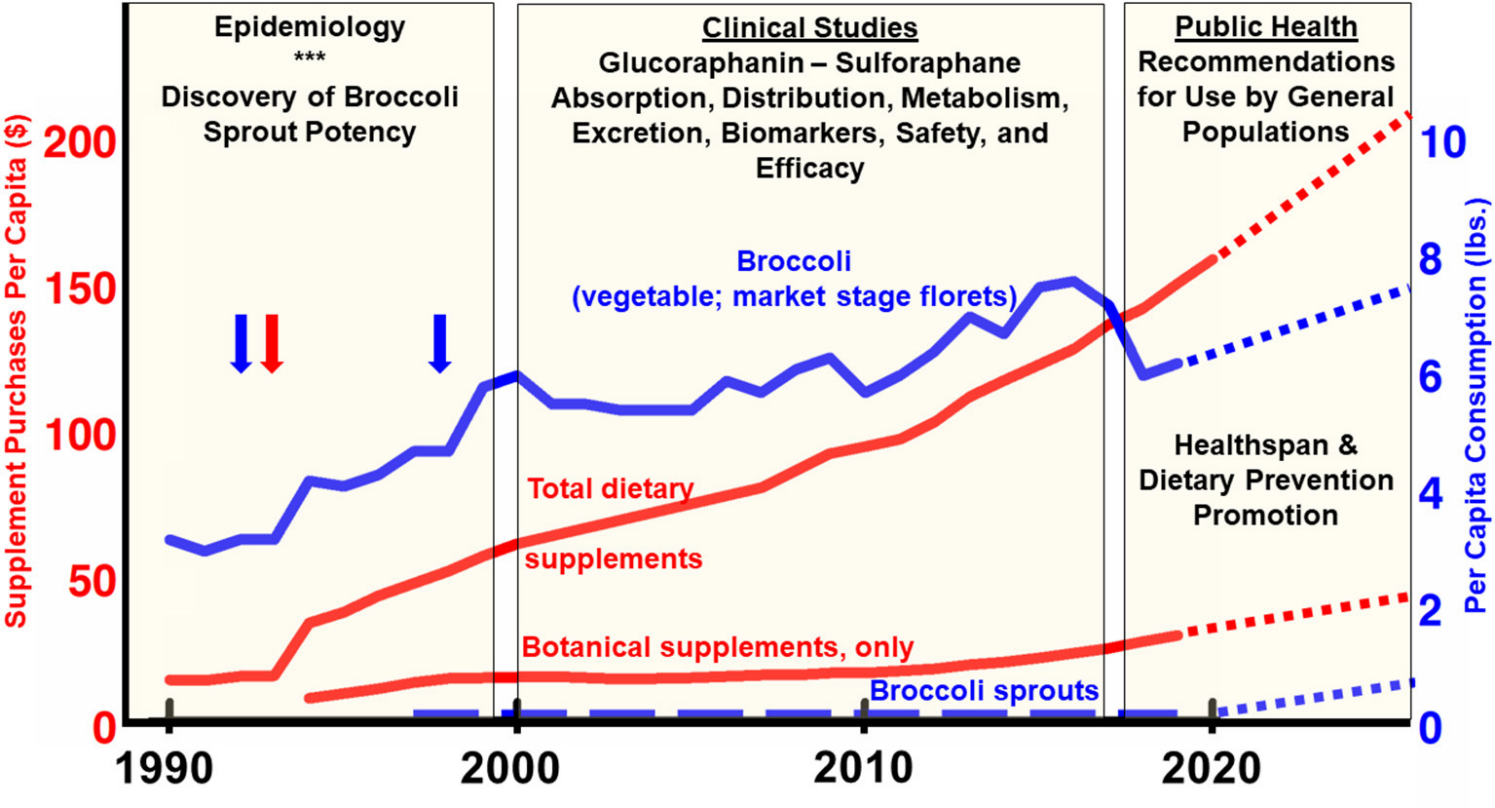
USA consumption per capita between 1990 and 2020. Broccoli per-capita consumption figures reported by USDA Economic Research Service in pounds (blue line; *right* axis) (103). Blue vertical arrows align with time of first discovery of sulforaphane and description of some of its benefits (3) and of broccoli sprouts (5). The red vertical arrow aligns with the 1994 enactment of DSHEA (the Dietary Supplement and Education Act) which provided a huge stimulus to the supplement industry. Broccoli sprout consumption is imputed, based on our best estimates since no reliable market data exists (dashed blue line; *right* axis). Since weight-or mass-based metrics would be a meaningless comparison for dietary supplements, total sales (dollar value), and botanical (herbal) supplement sales, are normalized to the US population over the time periods for which data was available (red lines; *left* axis) (104), and the botanical supplement data break-out came from the American Botanical Council (105, 106). Sales of glucoraphanin, sulforaphane, and broccoli seed- and sprout-rich extracts are a fraction of this magnitude, but no reliable market data exists. All dotted lines for times beyond 2020 are editorial “best guesses”.

Although a “new” food since 1997, broccoli sprouts are not intrinsically sensual. There are enough taste, shelf-life, and food-borne illness complications with them that they will likely never take off as a dominant part of the grocery shelf in the USA (see Figure 5). Home-sprouting, will likewise also never reach the masses. Broccoli seed and sprout extracts are not magic. They are still very clearly a work in progress, but that progress is presently driven by companies large and small, which are restricted in the claims they can make. Patenting phytochemicals from non-genetically engineered plants is prohibited, as well it should be; those companies do not have the profit incentive that drug companies clearly have. Thus, supplement companies operate in the unfiltered mass market (e.g., via Amazon, supermarkets, and drug stores), as well as through the alternative health practitioner space (e.g., by prescription from nutritionists, dieticians, naturopaths, and other alternative or complementary practitioners, but typically not from allopathic physicians). This leaves responsible companies who are marketing and producing similar products with identical active ingredients, to compete for market share by shaving cost of goods (inputs), performing minimal or no research, and making claims that come as close to the legal line as possible. and of course imaginative advertising and marketing. Irresponsible companies and fly-by night internet vendors can make huge profits with outlandish claims, get shut down or forced from the market by regulators, and pop up in unfettered fashion with another site/claim/product.

Where does this landscape leave the responsible use of a phytochemical that has an outstanding safety profile and a rosy future in at least some domains of prevention and therapy, as we hope we have illustrated herein? We see little suggestion that consumers' appetite for dietary (a.k.a. nutritional) supplements will abate, and every indication that it is increasing by single to even double-digit percentages annually, having grown into a $52 billion dollar industry by 2020 (Figure 5). We know that only a small fraction of that market encompasses botanicals (e.g., broccoli and its phytochemical components) (93). We see little evidence that sprouts will command an increased percentage of the typical household's food budget, nor are we advocating that. And we are likewise not advocating that every promising phytochemical—products of nature and lengthy plant evolution as they are—be cast as a drug, patented, and confronted with all of the sorts of trials and regulation that their synthetic and semi-synthetic cousins in pharma are subjected. Even with the lay press attention to broccoli as a “superfood,” as well as the abundance of scientific evidence that one might think would bolster its position on the dinner plate, the statistics over the past two decades support the narrative that per capita consumption of broccoli itself (florets of the horticultural commodity that have long been a popular or not-so-popular green vegetable) has barely changed at all (ca. 6 lb/person/year) (Figure 5).

Conversations about increasing the proportion of fresh vegetables in the average person's diet are not extremely controversial, nor is the statement that cruciferous vegetables or even broccoli or broccoli sprouts should be a part of that mix. It is a question for public health experts and policy-makers as to how to more effectively execute that increased presence of vegetables as a strategy for enhancing healthspan. However, the jury is very much still out about the wisdom of recommending dietary or nutritional supplements to otherwise healthy adults (or children). A very good case can be made that as we age out of our reproductive years, evolution has not taken very good care of us. Our needs for supplementary vitamins, minerals, and phytochemicals of various sorts increases if we want to maintain healthspan well into our personal second half-centuries. Clearly, many of us would not even be alive to worry about that if it were not for antibiotics, anti-arrhythmics, thyroid hormones, statins, and a list of other pharmaceuticals. Healthy diets such as the Mediterranean or Blue Zones diets likely enhance the quality of our trajectory to its inevitable end-point. Stepping back from the food and drug precipice, it should be noted that more than three quarters of all Americans now take supplements, and 10% of us take four or more such supplements (94–96). It is thus not heretical to assume that supplements might- and could-be part of the solution, should the science support such a strategy. It is important to note, however, that despite encouragement from epidemiological studies targeting phytochemicals, evidence to date from randomized clinical trials with mineral or vitamin supplements does not support efficacy for reduction of cancer risk (97). Perhaps more mechanism-based interventions with bioactive phytochemical supplements will show merit.

Much as we ourselves have been immersed in this research, we maintain that the potential of sulforaphane and phytochemicals like it have not been properly explored. The approach has, by definition, been all over the map: Researchers gratefully get funding wherever they can, but it is hardly ever sufficient to see the job through. More critically, it comes without the widespread policy focus that is needed to evaluate promising phytochemicals in isolation, and in their respective food matrices, and to bring them, and combinations of them, through well-funded and rationally designed clinical trials.

This therefore brings us to three very different, but complementary suggestions for the future of phytochemicals like glucoraphanin and sulforaphane and the plants in which they are found:

1. Spend more money imaginatively promoting and marketing healthy diets rich in fresh fruits and vegetables of all sorts. This applies both to the category in general, as well as to those specific foods with epidemiologic and mechanistic evidence behind them. This approach should first, however, be augmented by an accurate mapping of our “foodome”—our full chemical exposure via our diets—using advances in machine learning and chemical identification, as so presciently articulated by Barabási et al. (1). This is a societal fix that can be driven by schools of public health and state and federal health agencies, and need not exclude industry. It is not novel, and it has already stumped many of the best minds in that space although there of course have been small victories.
2. Enhance the regulation and oversight of the supplement industry. This presents a major opportunity to improve the quality and safety of the products of that industry (98), but even more importantly, the claims which they make and the evidence upon which those claims are based. This is in large part a regulatory fix, which means it is highly political. We are not particularly sanguine about the chances for a sea change here, but it must be considered.
3. Increase by at least an order of magnitude, funding for prevention research, and for science-based interventions. This increase in funding must include healthspan (healthy aging) and nutrition, and should include both the prevention and treatment of chronic illness. It is a mammoth undertaking and it will take political will and societal commitment. Funding could and should come from a variety of sources including the NIH, and the industries that have a stake in the outcome. which means the following sectors of society at a minimum: insurance, supplement, pharmaceutical, food, agriculture/farming, healthcare. in fact no sector of society is untouched or not affected and most sectors should or could conceivably become involved. Philanthropic foundations should certainly play a part, and to their credit many have already pitched in, but the job is still too mammoth and will require vision and leadership on a scale that we have not yet been able to muster. The mobilization of these communities into partnerships amidst the COVID-19 pandemic exemplifies what can be accomplished in response to an acute health crisis. More intense and sustained partnerships will be required to enhance healthy aging on a global scale.

The drug or pharmaceutical industry (“Pharma”) is not motivated to fund research into phytochemicals, except, and perhaps, as it pertains to small molecule delivery systems. Intellectual property (patents) are the bedrock of this industry, and natural products are by their very nature not patentable. However, Pharma does have tremendous incentive to modify existing molecular scaffolds to “improve upon” products of nature. They have historically done that extensively, and very well (99). A propos the thesis of this commentary, Pharma is working very hard to target the Nrf2 pathway, which is a primary target of sulforaphane (22, 70), and the cross-category utility of this research and even the results of some clinical studies done with these small molecule drugs may be great. Thus, there is potential that money from Pharma may benefit the science of sulforaphane in general. But prescribing drugs in order for large swaths of the population to remain healthy is not what we are advocating and care must be observed not to let the conversation move too far in that direction.

“Farma”—agricultural, farming, and food enterprises collectively—serve to profit when value-added food products are developed. If these are whole fresh foods (e.g., new and improved vegetable varieties with enhanced and standardized phytochemical content such as higher glucoraphanin broccoli for seeds or sprouts or florets), it is clear that not only the farmers who grow those varieties, but supermarkets, seed companies, agricultural chemical companies, processors, farm equipment vendors, and other middlemen could all benefit. If these added value products span the range between minimally- and highly processed foods—an inescapable part of modern existence—large multinational food companies and fast food operators would additionally stand to reap financial benefits, as would freeze-drying, flavoring, and other food-associated businesses. It is thus hard to imagine an industrial sector that would not profit from increased sale of healthy foods, yet historically these industries have not been supporters of the research required to develop such healthy foods. Excellent arguments have been made that various manifestations of Farma (as defined above) will have to be part of the solution even though they are clearly also part of the problem.

In the USA, federal funding of the research underpinning phytochemicals such as sulforaphane has been out of proportion with their influence on maintenance of long term health. Thus, their value as preventive dietary agents gets very little attention, until and unless a specific disease indication is identified, at which point they become potential therapeutics and are suddenly perceived as being more deserving of research funding. The burden of funding plant breeding and horticultural research has largely been left to the USDA, which has a mission that is perceived by some to be at odds with the goal of developing healthy foods due to the obeisance of this agency to the meat and dairy industries. Some NIH funding has gone to sulforaphane, resveratrol, curcumin, and a small number of other phytochemicals, but funding for the basic biology related using phytochemicals to support healthspan or prevention, has been woefully inadequate. This may reflect the fact that mainstream nutrition hardly even acknowledges phytochemicals, and of course physician training includes almost no nutrition! As example of this lack of attention, in over 100 pages of the National Academy of Science's “Advancing Nutrition and Food Science: 80^th^ Anniversary of the Food and Nutrition Board,” there is not a single acknowledgment of phytochemicals or any permutations of that word and topic (100); nor is there, in the 64-page white paper entitled “The Lancet Commission on diabetes: Using data to transform diabetes care and patient lives” (101).

The dietary supplement industry as it is presently construed appears to have the greatest incentive to fund such research. By and large they have not done so, because they are still growing at 6–7% per year, and they don't yet have the urgency to do so. That said, they are keenly aware that successful clinical studies conducted with supplements (phytochemical-based, as well as vitamins, minerals, botanicals, nutritional supplements such as creatine, pea protein, and the entire spectrum of products of that industry) advance the cause of their specific products, and advance the profile of the industry in general. With little or no intellectual property on the compounds themselves, and only some on the delivery systems, their opportunities for exclusivity with any specific phytochemical is very limited, and thus they do not invest in the kind of mechanistic work that is so critical, though they do invest in methodologies for phytochemical delivery systems.

And lastly, economists are very much needed to focus on prevention and healthspan. This is already being done across the globe by schools of public health, funded by a variety of philanthropies as well as the NIH. The levels of support are trivial, though, compared to what is required to stem the growing and inevitable assault on our economy due to a more and more unhealthy, aging population. Two of the ten largest industries in the US (health and medical insurance) are logical targets for not only financial support, but for imaginative and creative thinking about solutions. That they are already thinking in this direction is clear and obvious (102); however, the phytochemical and biomedical research community is not well-connected to these potential sources of both funding and ideas. It is quite clear that with our aging population, poor diets, and expensive drugs and healthcare, our healthspan cannot tolerate business as usual. Phytochemicals such as sulforaphane from broccoli sprouts should be part of a re-invention of our healthcare.

## Data Availability Statement

The datasets analyzed for this review are found in the cited, published literature.

## Author Contributions

JWF and TWK contributed to all aspects of this article and agree to be accountable for the content of the work.

## Conflict of Interest

The authors declare that the research was conducted in the absence of any commercial or financial relationships that could be construed as a potential conflict of interest. JWF has consulted for both food and supplement companies in the past year.
